# Directing Cholangiocyte Morphogenesis in Natural Biomaterial Scaffolds

**DOI:** 10.1002/advs.202201951

**Published:** 2022-05-16

**Authors:** Quinton Smith, Jennifer Bays, Linqing Li, Haniyah Shareef, Christopher S. Chen, Sangeeta N. Bhatia

*Adv. Sci*. **2022**, *9*, 2102698

DOI: 10.1002/advs.202102698

Several key pieces of information are inadvertently missing in the original manuscript. Please find below the updated Figure 4 caption in which the gel conditions are given and a new method detail, that describes the assembly and seeding protocol for the microfluidic device used in the manuscript. The authors apologize for any inconvenience this may have caused.

**Figure 4. Intrahepatic Biliary Ducts on a Chip. A)** Immunofluorescence images, characterizing marker expression and morphology of normal rat cholangiocytes grown on planar 2D surfaces and in 3D type 1 collagen gels (3 mg mL^−1^). **B**) Experimental schematic describing the co‐culture of J2 fibroblasts and NRCs separated by a collagen gel. In addition, representative confocal images of NRCs after 9 days of culture are shown (with NRCs cultured at low density [20,000 cells per well] and high density [50,000 cells per well]), highlighting the ability to form branched lumenized structures with stromal support (n = 3 gels). **C**) (i) Device schematic and representative brightfield images, showing top and cross‐sectional views of a dual channel microfluidic platform; (ii) encased within the biomaterial scaffolds are two parallel 300 µm open lumenal structures spaced 1 mm apart; (iii) the arrangement provides capacity to seed IHCs and flow media in the patent channels; (iv) and encapsulate IHCs in the bulk compartment of the device. **D)** Representative time course images of LifeAct RFP‐IHCs grown in microfluidic device with 20 ng mL^−1^ EGF, showing collapse of open structures after six days, but anastomosis between bulk networks with channel structures. **E)** Representative maximum intensity projection images of LifeAct RFP‐IHCs cultured for 6 days with 20 ng mL^−1^ EGF in 5 mg mL^−1^ fibrin scaffolds, showing maintenance of open cell‐laden channels compared to Matrigel/collagen type 1 scaffolds (Nuclei stained with DAPI). **F)** Representative brightfield image of microfluidic cultured NRCs, grown over the course of 7 days (n = 3 devices). **G)** Confocal z‐projection alongside an inset **H)** of microfluidic cultured NRCs showing polarized epithelium along the channel walls (n = 3 devices).

**Biliary Network Formation**. Collagen gels were formed as previously described. In brief, 1.0 × 10^6^ cells mL ^−1^ were encapsulated in gels composed of Matrigel mixed at equal parts with 3.0 mg mL ^−1^ rat tail collagen type I (Corning), that was titrated to pH 7.0‐7.5 with 1M NaOH. 100 µl of the collagen mixture was added to wells of a 96 well plate and allowed to polymerize at 37°C for 30 minutes. For fibrin gels, 1.0 × 10^6^ cells mL^−1^ were resuspended in fibrinogen (5 mg mL^−1^; Sigma) and crosslinked with thrombin (3 U mL^−1^; Sigma). An additional 100 µl of epithelial media was added to the gels after solidification.

**Microfluidic Device Assembly**. Two‐channel microfluidic devices were fabricated as previously described^[1]^. In short, tubular channels were formed in the PDMS devices, after functionalizing the surfaces with 0.01% poly‐l‐lysine and 1% glutaraldehyde to promote ECM adhesion. Two 300 µm diameter acupuncture needles (Hwato), blocked with 0.1% (w/v) bovine serum albumin (Sigma), were inserted into the needle guides of the devices. ECM solutions (collagen, collagen/Matrigel or fibrin) containing cells, were cast into the device, and allowed to polymerize for 30 minutes at 37°C. Needles were removed following gel polymerization and seeded with cells to line the channels. In experiments with NRCs, channels were coated with 50 µg mL^−1^ laminin for at least 1 hour at 37°C following needle removal.

